# Computational Resources in Infectious Disease: Limitations and Challenges

**DOI:** 10.1371/journal.pcbi.1000481

**Published:** 2009-10-26

**Authors:** Eva C. Berglund, Björn Nystedt, Siv G. E. Andersson

**Affiliations:** Department of Molecular Evolution, Uppsala University, Uppsala, Sweden; Massachusetts Institute of Technology, United States of America

Infectious diseases continue to be a major cause of death in the human population, with tuberculosis and malaria affecting 500 million people and causing 1–2 million deaths annually [1]. The situation is aggravated by the increasing prevalence of antibiotic-resistant bacteria and the risk that terrorists might use infectious organisms to aggress target populations. During the past decade, we have also witnessed the emergence of many new pathogens not previously detected in humans, such as the avian influenza virus, severe acute respiratory syndrome (SARS), and Ebola. The appearance of these novel agents and the reemergence of previously eradicated pathogens may be associated with the growing human population, flooding, and other environmental perturbations; global travel and migration; and animal trade and domestic animal husbandry practices. Simultaneously, we have seen an explosion of genome sequence data. Sequencing is now the method of choice for characterization of new disease agents, as exemplified by the rapid sequencing of the genome of the SARS virus, which was made available within a month of identification of the virus [2],[3]. Like SARS, most newly emerging disease agents originate in animals and have been transmitted to humans recently at food markets, by insect bites, or through hunting [1].

The new sequencing technologies enable small academic research groups to create huge genome datasets at low cost. As a result, scientists with expertise in other fields of research, such as clinical microbiology and ecology, are just beginning to face the challenge of handling, comparing, and extracting useful information from millions of sequences. Here, we discuss the limitations of publicly available resources in the field of genomics of emerging bacterial pathogens, emphasizing areas where increased efforts in computational biology are urgently needed.

## Genome Evolution in Emerging Bacterial Pathogens

**Figure 1:**
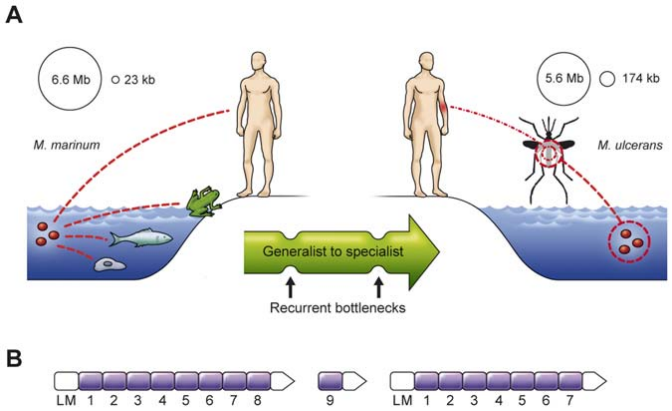
Evolution of a new infectious disease agent. (A) Recent evolution of the specialist human pathogen *M. ulcerans* from the aquatic generalist pathogen *M. marinum*. (B) Arrangement of the three *M. ulcerans* plasmid–encoded repeated virulence genes (arrows from left to right: *mlsA*1 [51 kb], *mlsA*2 [7.6 kb], *mlsB* [43 kb]) coding for three polyketide synthases. The loading modules (labeled LM) and the 16 repeated modules depicted in purple (labeled 1–9 for *mlsA*1 and *mlsA*2, and 1–7 for *mlsB*) enable the serial buildup of the backbone carbon chain of the complex immunosuppressive substance mycolactone.

A natural ecosystem of a bacterial population that incidentally infects humans provides a high-risk microenvironment for the establishment of this pathogen in the human population (Box 1; Figure 1). Comparative studies of the genomes of well-recognized human pathogens, incidental pathogens, and their closely related nonpathogenic species [4]–[11] are valuable for efforts to predict the propensity for host shifts and their consequences for human health.

**Box 1. Genomic Changes Associated with Host Shifts**

The movement of a bacterial species from abundant animal hosts such as rodents, which are a major reservoir of infectious disease agents, to the relatively small human population is typically associated with decreased genome size and loss/alteration of the mobile gene pool [4], [32]–[34]. One illustrative example can be found in the genus *Mycobacterium*, which contains several severe human pathogens, including the agents of tuberculosis (*M. tuberculosis*) and leprosy (*M. leprae*) and also the recently emerged *M. ulcerans*. *M. ulcerans* causes severe skin lesions; this disease, known as Buruli ulcer, is becoming a serious public health problem in West and Central Africa as well as in other parts of the tropics.

Like many other recently emerged human pathogens [4], [34]–[36], *M. ulcerans* appears to have switched from a generalist to a specialist lifestyle: starting with a progenitor very similar to the aquatic *M. marinum*. While *M. marinum* has been found both free-living and as an intracellular pathogen of fish and other species, *M. ulcerans* is thought to have a restricted host range and to be transmitted by insects (Figure 1A). The host switch was likely initiated by the uptake of a virulence plasmid, and preceded through a series of “bottleneck events” or (severe reductions in population size due to environmental circumstances). This process resulted in loss of about 1 Mb of the genome, major genomic rearrangements, extensive proliferation of insertion sequences, and a massive increase in number of pseudogenes [37]–[39]. In particular, there was a massive reduction in the size of the two major surface protein gene families (a decrease of more than 250 genes compared to *M. marinum*). This gene loss is thought to have been crucial for the organism to evade the human immune system, by limiting the number of antigens on the bacterial surface [40].

The uptake of a new virulence plasmid producing an immunosuppressive substance called mycolactone is also thought to have played a key role in the evolution and host switch of *M. ulcerans*. This plasmid consists mainly of three unusually large and internally repeated genes (over 100 kb in total), and thus illustrates the concept of long and repeated virulence genes (Figure 1B) [41]. These genes appear to evolve rapidly by recombination and gene conversion, and new variants can be directly connected to variations in the chemical structure of mycolactone [42], which might be important for host specificity, immunosuppressive potency, and drug design.

A successful infectious bacterium, whether it causes disease or not, must possess mechanisms for interacting with the host and evading the host immune system. The key players in these processes are often proteins on the surface of the bacterium, including secretion systems that release effector proteins into the surrounding medium or directly into the host cells. These host-interaction factors are often members of large protein families with many paralogs and often encoded by long genes with internal repeats. Fluctuations in gene length and copy number occur through homologous recombination over these repeats [12]–[15].

Adding to the variability of the host-interaction genes is that they are often located on mobile elements such as plasmids or bacteriophages, which are easily gained and lost. Rapid sequence evolution of these genes may be driven by selection, because it often increases bacterial fitness by escaping the host immune system, creating a diverse set of binding structures or tuning effector proteins to a new host. As a consequence, host-interaction genes typically show extreme plasticity in both sequence and copy number, partly because they are under strong evolutionary pressure and partly because they are mechanistically prone to drastic mutational changes. Understanding these complex dynamics poses major challenges in many areas of computational biology, ranging from sequence assembly to epidemic risk assessment.

## Complete Genome Assembly Remains Difficult

Despite the ease with which shotgun sequence data can be generated, assembling these data into a single genomic contig remains labor-intensive and time-consuming. This obstacle is primarily due to the difficulty of assembling repeated sequences. Hence, resequencing approaches—where short sequence reads are directly mapped to an already completed reference genome—have become increasingly popular. Resequencing readily detects SNPs (single nucleotide polymorphisms) in single-copy genes, but performs very poorly in repeated and highly divergent regions of the genome. Genes involved in infection processes, with their complex repeat structures, high duplication frequency, and rapid evolution, are thus often left unresolved.

The perhaps most imminent need is not for improved assembly algorithms but for better ways to integrate data from diverse sources, including shotgun sequencing, paired-end sequencing, PCR experiments, fosmid and BAC (bacterial artificial chromosome) clone sequencing, physical mapping, and restriction fragment data. A program integrating these different data should not only accurately assemble as much of the genome as possible, but also assist the researcher in designing additional experiments to resolve the remaining regions. Given the rapidly increasing number of incomplete genome sequences available, it would also be valuable with a quality-scoring standard that not only provides quality scores at individual sites under the assumption that the assembly is correct, but also reflects the uncertainty of the actual assembly over specific regions.

While assembly software development is struggling to keep up, the sequencing revolution shows no signs of slowing down. Perhaps the most important new development is real-time single molecule detection platforms with ultra-long sequencing reads [16]. Within the next few years, we can expect to see read lengths of 20 kb, which will help resolve many of the complex genomic features underlying host adaptation and pathogenicity.

## Functional Annotation of Virulence and Host-Interaction Genes

Annotation is the process of assigning meaningful information, such as the location or function of genes, to raw sequence data. Reliable and consistent annotations are thus fundamental for analysis and interpretation of genome data. Since annotation of new genomes is usually based on homology searches (e.g., BLAST hits), errors and inconsistencies tend to propagate. One way to reduce error propagation is to functionally annotate a set of reference genomes based on experimentally determined information. Annotation of new genomes could then start with searches in this database, which would allow high-quality annotation of all well-conserved genes. The Gene Ontology's Reference Genome Project [17] and BioCyc [18] represent developments in this direction. However, the number of species included is still limited, and a broader taxonomic breadth of bacteria, with one reference species per genus, would be desirable.

Functional annotation of pathogen genomes is particularly important, because genes involved in host-interaction processes are among the most difficult to annotate. One problem is that different research groups often have studied homologous genes in various species, and given them different names that are not always logical or reflective of similarities in sequence and function. A manually curated database of protein families involved in host interactions that incorporates currently used gene names, sequence motifs, gene functions, and experimental results would substantially improve the situation. Much improved guidelines for how to annotate genes in large families with different combinations of sequence motifs would also be valuable.

**Figure 2:**
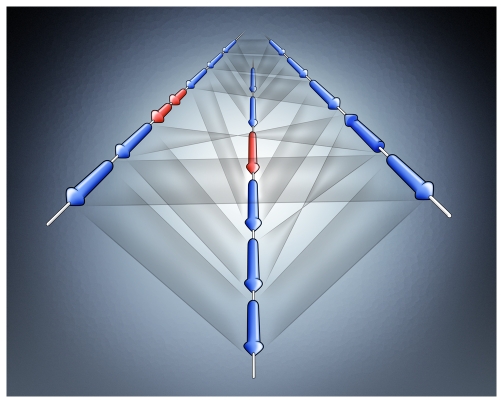
New visualization tools for genome comparisons. Comparison of the genes in multiple genomes can be represented visually by using a 3D program. Each arrow represents one gene, and the grey shading between genes indicates homology. Red indicates genes that are unique to one genome. The difference between this approach and existing programs is that all genomes can be compared to each other simultaneously, rather than by pairwise comparisons. With multiple genomes, and with zooming, flipping, and selecting options, even this rudimentary 3D program would be of great help in genome analysis.

Comparative studies of very closely related genomes can help to distinguish functional genes from spurious ORFs (open reading frames) and pseudogenes, and thereby improve gene prediction. To this end, a tool to visualize all the fine details in comparisons of multiple closely related genomes is crucial. Such a tool was developed recently for genomes with a conserved order of genes, and it has been applied to analyze sequence deterioration in the typhus pathogen *Rickettsia prowazekii* and its closest relatives [10]. Future studies, however, will require software that can also handle multiple genome comparisons from highly rearranged genomes. Another limitation of currently available visualization tools is that, although multiple genomes can be included, only serial pairwise comparisons can be made. This limitation can be overcome by visualization of genome comparisons in “three dimensions” (3D visualization), enabling all-against-all comparisons to be viewed simultaneously (Figure 2). Just as 3D visualizations revolutionized the field of structural biology over the past decades, such developments might well revolutionize the field of comparative genomics in the years to come.

## Molecular Diagnostics and Vaccine Development

Classification of infectious disease agents is typically based on multilocus sequence-typing (MLST) systems, by which new bacterial isolates are analyzed by sequencing five to seven predefined core genes [19]. With the increasing number of complete genome sequences of pathogenic and nonpathogenic strains, it will be possible to concatenate a much larger number of conserved genes and use this dataset to infer a tree to represent the underlying population structure [20]. However, while genotyping systems based on conserved genes can be useful for monitoring the spread of strains, they do not necessarily correlate with genomotypes defined by virulence properties [21]. This is because genes contributing to virulence are prone to horizontal gene transfer, gene duplications, and gene loss. Further complicating the development of molecular diagnostic methods is that homologs of virulence genes are often present also in nonpathogenic species, making it difficult to recognize pathogens solely from the gene content. Hence, classification and risk assessments for the emergence of novel infectious strains ultimately should be based on a combination of strain typing, gene content, and identification of virulence genes.

**Figure 3:**
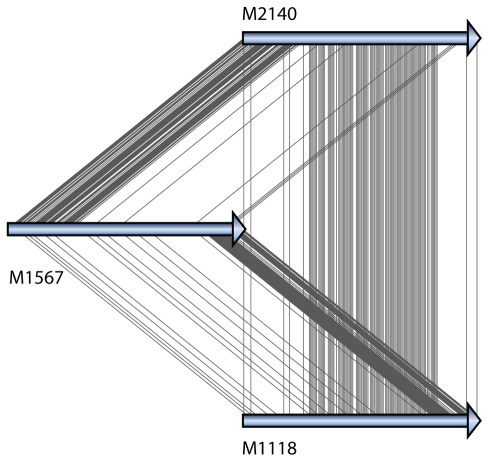
New methods for analyzing evolution by recombination. Improved models and visualization tools are needed to analyze recombination. Virulence genes, here exemplified by the *acfD* gene in the *Vibrio cholerae* pathogenicity island [43], often display complex recombination patterns. The aligned *acfD* genes (arrows) from three *V. cholerae* strains (M2140, M1567, and M1118) are plotted separately; a line connects each site where the nucleotides in two strains differ from the third strain. Noninformative sites were removed before plotting.

Understanding the evolutionary dynamics of host-interaction genes in terms of both mechanisms and selective forces is also important in order to design drugs that will be effective in the long term. What good would be the development of a new antibiotic or vaccine if the intended target protein evolves beyond recognition before the drug reaches the market? One solution to this problem is to characterize the selective pressures on candidate vaccine targets, and then exclude genes or parts of genes based on their evolutionary dynamics [22]. However, current tools for measuring positive or diversifying selection are severely limited in that they assume that single-base mutations are the only underlying mechanism of sequence change. For reliable analyses of genes with a complex evolution, a new generation of evolutionary tests needs to be developed that acknowledge the importance of mutation by recombination (Figure 3) and multiple-base insertion/deletion events as well as point mutations. With the expected huge increase of complete and draft genomes for many strains of a species, there is a need for programs capable of screening a large set of alignments for recombination signals, with novel statistical and visualization tools to analyze the full set of results.

## Predicting Risk for Disease Outbreaks

The next challenge is to place the genomic data within its ecological context, which has led to a new research field called molecular ecosystems biology [23]. This field focuses on dissecting the many complex molecular interactions between the bacterial population and its environment. This environment can be highly specialized, as in the case of bacteria adapted to a single host species, or very complex as for soil-, water-, or airborne bacteria. The behavior of a pathogen thus depends on many ecological factors, such as seasonal fluctuations in temperature and nutritional availability, species richness and host population density.

To be able to integrate and evaluate these data, new software is needed. Imagine a program that can read sequence data from hundreds of bacterial isolates, infer the underlying population structure, and combine it with gene expression data, ecological factors, and clinical data such as the number of disease cases reported in various geographic areas. It should be possible to visualize global patterns in the data, such as abundance of particular strains and sequence variants and migration of infected hosts and vectors over geographic areas and seasons. Changes in taxonomic profiles, virulence genes, and metabolic pathways should be visualized in real time. This program could also be linked to a Web site where researchers can post daily updates of clinical cases, spread of virulence genes, appearance of new strains and new mutations, migration patterns, and news about genome and functional data. This site would be useful for estimating the risk for new epidemics to emerge in the human population.

## Analyzing Microbial Communities

Analyzing the behavior of complete pathogen ecosystems is an immediate priority. Random shotgun sequencing projects of bacterial DNA from diverse environments count in the hundreds, and the amount of metagenomic sequence data already exceeds the available genomic sequences in public databases [24],[25] (http://www.genomesonline.org). Several multinational projects on the human microbiome have been launched, which, together with studies of 16S rRNA amplicons, have provided new insights into the human intestinal [26]–[28], oral [29], and vaginal flora [30]. Comparison of the microbial flora in healthy and diseased people can be a powerful diagnostic tool and enable the discovery of both emerging pathogens and novel virulence factors, such as antibiotic resistance plasmids. An important technical development that holds great promise for associating the functional adaptation of the community as a whole with the metabolic pathways present in the individual strains is single-cell isolation followed by whole-genome amplification. Community sequencing also provides an excellent tool for epidemic surveillance of pathogenic strains and virulence genes in environments from which they may further spread to humans.

The massive amount of data created by microbial community sequencing poses new challenges and will require extensive bioinformatics development [24]. Although the advent of longer sequence reads will have a large impact on the assembly of community data, the presence of many closely related species or strains in the same sample, along with horizontal gene transfer, will remain a daunting challenge. A whole new field of comparative algorithms needs to be developed, for example to provide meaningful comparisons between taxonomic profiles. New sequence databases will be essential for rapid access to both raw and processed data. Also, for fair comparisons between datasets, a certain level of standardization of sampling, experimental work, and statistics will be crucial [31]. Bioinformatics skills combined with a deep biological understanding of the system under study are needed to use these complex sequence datasets to answer such questions as: Who is there? What are they doing? How are they communicating? And what is the risk for disease?

## Challenges for the Future

The priority goals for the next decade within the area of emerging infectious diseases should be the study of complete pathogen ecosystems and the dissection of host–pathogen interaction communication pathways directly in the natural environment. To achieve these goals, investments in user-friendly software and improved visualization tools, along with excellent expertise in computational biology, will be of utmost importance. Unfortunately, too few undergraduate students in clinical microbiology and microbial ecology are trained in computational skills, and national governments and universities need to take action to address this deficiency to meet the demands of the near future. Often neglected by public and private funding is the monumental need for stable and standardized infrastructure at all levels, from the individual research group to the intergovernmental organization. Only with proper investments in everything from hardware and personnel for data handling, to the development of sensible and standardized file formats, can we ensure that the current developments can be fully exploited to more efficiently battle emerging infectious diseases.

Currently, the slow transition from a scientific in-house program to the distribution of a stable and efficient software package is a major bottleneck in scientific knowledge sharing, preventing efficient progress in all areas of computational biology. Efforts to design, share, and improve software must receive increased funding, practical support, and, not the least, scientific impact. Since microorganisms do not follow national borders, such initiatives are probably best started from intergovernmental organizations with close links to national centers with established communication networks to distribute know-how and advances further within the country, and vice versa, to facilitate the spread of new concepts and software to all members of the organization. Eventually, many of these initiatives may become community-driven. The example of Wikipedia, with more than 10 million entries written since the launch in 2001 and a current growth rate of thousands of articles daily (http://www.wikipedia.org), demonstrates the power of user-contributed initiatives.
